# Siglec-15: a potential regulator of osteoporosis, cancer, and infectious diseases

**DOI:** 10.1186/s12929-019-0610-1

**Published:** 2020-01-03

**Authors:** Takashi Angata

**Affiliations:** Institute of Biological Chemistry, Academia Sinica, 128, Section 2, Academia Road, Nankang District, Taipei, Taiwan

**Keywords:** Siglec-15, Sialic acid, Osteoclast, Macrophage, Cancer, Microbial infection

## Abstract

Siglec-15 is a member of the Siglec family of glycan-recognition proteins, primarily expressed on a subset of myeloid cells. Siglec-15 has been known to be involved in osteoclast differentiation, and is considered to be a potential therapeutic target for osteoporosis. Recent studies revealed unexpected roles of Siglec-15 in microbial infection and the cancer microenvironment, expanding the potential pathophysiological roles of Siglec-15. Chemical biology has advanced our understanding of the nature of Siglec-15 ligands, but the exact nature of Siglec-15 ligand depends on the biological context, leaving plenty of room for further exploration.

## Introduction

Many glycan-recognition proteins (collectively called lectins) are expressed on leukocytes, and participate in self/non-self recognition and immune regulation. A family of sialic acid recognition proteins called Siglecs (an acronym for sialic acid + immunoglobulin superfamily + lectins) are expressed on various leukocytes, and modulate immune responses by recognizing ligands at the extracellular domain and mediating signal transduction at the intracellular domain [1, 2]. Involvement of Siglecs in various diseases, in particular cancer [3–7] and infectious diseases [8–14] are highlighted by recent studies. Whereas the majority of Siglecs interact with protein tyrosine phosphatase SHP-1 and suppress cell activation, a small fraction of Siglecs signals through adapter protein DAP12 (gene symbol: *TYROBP*) and tyrosine kinase SYK, activating (instead of suppressing) the immune cells that express them. Siglec-15 (gene symbol: *SIGLEC15*) is a member of the latter subfamily. Studies by several groups have revealed basic molecular properties of Siglec-15, its role in osteoclast differentiation, and more recently, its potential roles in cancer and in microbial infection. In this review, I will briefly summarize the works relevant to biological functions of Siglec-15 and the studies aiming at identifying Siglec-15 ligands by chemical biology approaches. (Note: gene symbols in human and mouse are italicized, and all letters are capitalized for human genes, whereas only the first letter is capitalized for mouse genes.)

### Molecular properties of Siglec-15

**Fig. 1:**
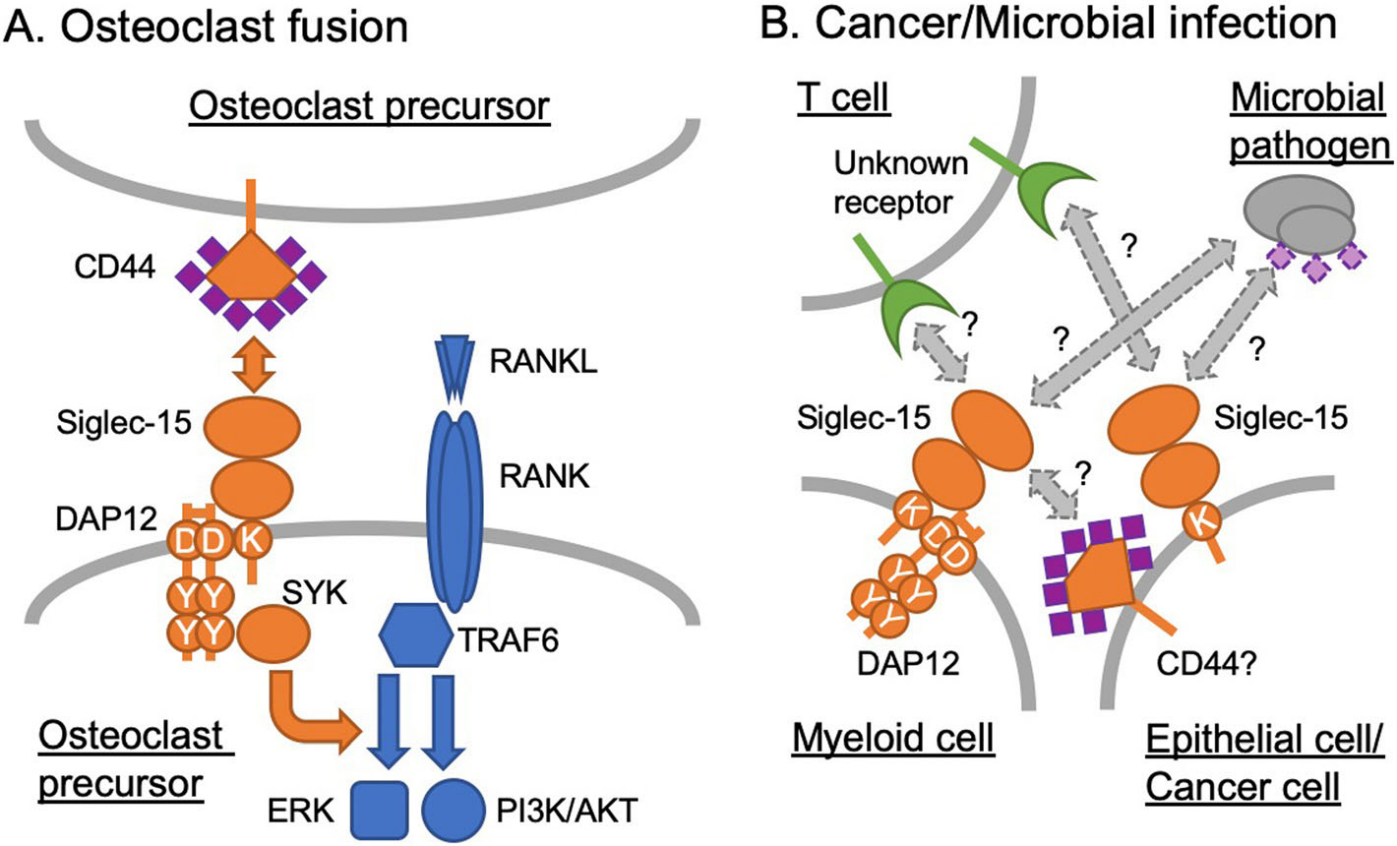
Models of Siglec-15–ligand interaction and downstream signaling. **a** Osteoclast differentiation. Siglec-15 on osteoclast precursor recognizes CD44 on adjacent osteoclast precursor and transduces the signal via DAP12–SYK pathway, which cross-talks with RANK–TRAF6 pathway and enhances downstream signaling (e.g., ERK and PI3K–AKT). Sialic acids (shown in purple diamonds) are required for this interaction. **b** Tumor microenvironment and microbial infection. In tumor microenvironment, Siglec-15 (on tumor-associated macrophages and/or cancer cells) engages an unknown receptor on T cells and dampens T cell responses required to suppress cancer growth. Likewise, Siglec-15 on myeloid and/or epithelial cells, induced by microbial pathogen, interacts with an unknown receptor on T cells and dampens T cell responses required to control infection. It is unknown whether the glycan recognition and/or signal transduction property of Siglec-15 is required in this model (Siglec-15 = ligand). Alternatively, Siglec-15 on myeloid cells may interact with cancer- or microbe-associated ligand and modulate the myeloid cell production of anti-inflammatory cytokine (e.g., TGF-β or IL-10), which suppresses T cell activation. This alternative model is similar to the one shown in (A) (Siglec-15 = receptor)

The human genomic DNA sequence corresponding to the N-terminal immunoglobulin-like domain of Siglec-15 was first reported in 2001 [15], and the full-length cDNA of human Siglec-15 was cloned by a Japanese consortium aiming at the comprehensive cataloguing of human transcripts [16]. The first molecular characterization of Siglec-15 was reported in 2007 [17]. Siglec-15 has an extracellular domain consisting of two immunoglobulin-like domains, followed by a transmembrane domain that contains a lysine residue (Lys274 in human Siglec-15) that is essential for the interaction with adapter protein DAP12, and a cytoplasmic tail (Fig. 1a). DAP12 has a very short (< 20 amino acids) extracellular domain followed by a transmembrane domain that contains an aspartic acid residue (Asp50 in human DAP12) and a cytoplasmic tail that contains a sequence motif called immunoreceptor tyrosine-based activating motif (ITAM), which recruits SYK upon phosphorylation. The interaction between Siglec-15 and DAP12 is based on the ionic bond at the transmembrane domains, as is the case with many other receptors that associate with DAP12.

**Fig. 2:**
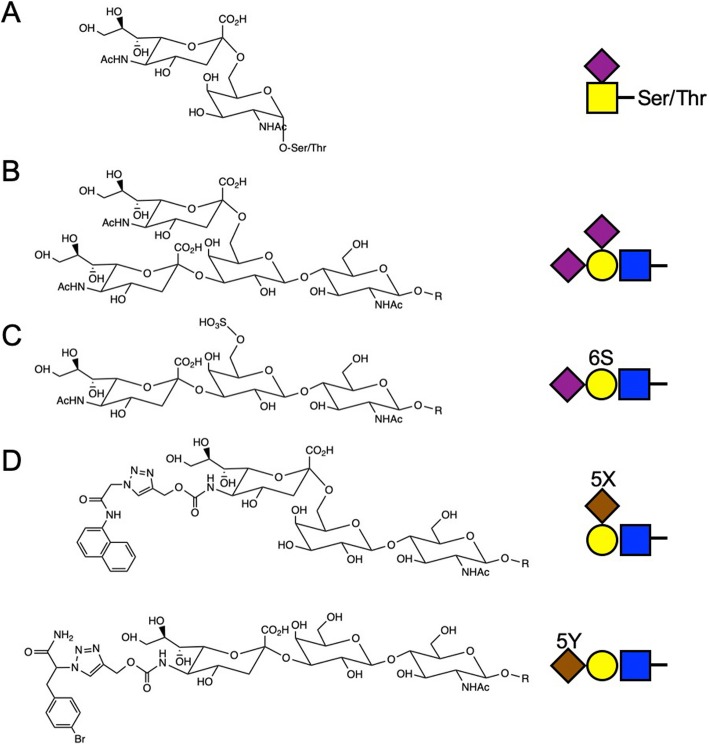
Glycan structures preferentially recognized by Siglec-15. **a** Sialyl-Tn (Neu5Acα2–6GalNAcα1-). **b** Non-natural glycan structure (Neu5Acα2–3[Neu5Acα2–6]Galβ1–4Glc/GlcNAcβ1-) preferentially recognized by Siglec-15 in Wu et al. [18]. **c** Sialylated and sulfated glycan structure (Neu5Acα2–3[HSO_3_–6]Galβ1–4GlcNAcβ1-) preferentially recognized by Siglec-8 [19]. **d** Non-natural glycan structures preferentially recognized by Siglec-15 in the study of Briard et al. [20]. Shown on the right are symbolic representations based on the Symbol Nomenclature for Glycans [21]

Siglec-15 was shown to bind preferentially to sialyl-Tn (Neu5Acα2–6GalNAcα1-; Fig. 2a) structure [17], although the variety of glycan structures used in the study was very limited. The glycan binding activity of human Siglec-15 was much weaker than that of mouse Siglec-15. Siglec-15 associated with an adapter protein DAP12, and also showed weak interaction with another adapter protein DAP10 in an artificial experimental system (over-expression of Siglec-15 and DAP10 in 293 T cell line); however, in vivo relevance of the latter finding is unknown. Using polyclonal antibody, Siglec-15 was found to be expressed in a subset of the cells that express DC-SIGN (a macrophage/dendritic cell marker) in human spleen and lymph nodes [17]. These findings implied that Siglec-15 may play a role in myeloid cells, but the in vivo role of Siglec-15 was unknown. The breakthrough discovery was brought about by several groups that independently revealed the role of Siglec-15 in osteoclast differentiation.

### Siglec-15 in osteoclast differentiation

Osteoclasts are multi-nucleated cells of myeloid lineage involved in bone resorption and remodeling. During bone remodeling, homeostasis is maintained by the resorption (bone breakdown) activity of osteoclasts, and the ossification (new bone formation) activity of osteoblasts. Osteoclast differentiation is primarily driven by the receptor activator of NF-κB (RANK) signaling pathway, which is triggered by the binding of RANK ligand (RANKL) produced by osteoblasts [22]. Osteoclast maturation requires auxiliary signaling through adapter proteins DAP12 and/or FcRγ [23, 24], and several receptors on osteoclasts (e.g., TREM2 [25, 26] and OSCAR [27]) were found to interact with these adapter proteins and participate in osteoclast differentiation.

The involvement of Siglec-15 in osteoclast differentiation in vitro was reported by two groups [28, 29]. Hiruma and colleagues [28] identified *SIGLEC15* as a gene highly expressed on giant cell tumor of bone, which resembles osteoclasts. By using polyclonal antibody against Siglec-15, they demonstrated that the antibody suppressed osteoclast differentiation of RAW264.7 mouse macrophage cell line (often used as an osteoclast precursor model), mouse bone marrow macrophages, and human osteoclast precursors [28]. Working independently, Ishida-Kitagawa and colleagues [29] found that Siglec-15 mRNA transcription is upregulated by transcription factor NFAT2, which is activated by RANK signaling. They demonstrated that Siglec-15 associates with DAP12 and signals through SYK, and the recognition of sialylated ligand by Siglec-15 is essential for osteoclast differentiation.

These in vitro findings were soon verified in vivo using genetically modified mice. Hiruma and colleagues reported that *Siglec15* null mice showed mild osteopetrosis (increased bone mass) in trabecular bones (i.e., porous, spongy bones) and reduced urinary deoxypyridinoline (a systemic marker of bone resorption), indicating reduced osteoclast activity [30]. However, the number of osteoclasts expressing lineage-specific marker (tartrate-resistant acid phosphatase, TRAP) was not reduced in the null mice. Takahata’s group, using another line of *Siglec15* null mice, demonstrated that their null mice also show mild osteopetrosis in trabecular bones [31], confirming the findings by Hiruma’s group. They further reported that the number of osteoclasts per bone surface was similar between wild-type and *Siglec15* null mice at primary spongiosa, whereas that at the secondary spongiosa was lower in the null mice (primary and secondary spongiosa represent different stages of bone calcification on cartilage, with the latter being more advanced). The difference between the primary and secondary spongiosa is explained by the presence of another ligand–receptor system (i.e. collagen–OSCAR•FcRγ [27]) promoting osteoclast differentiation in primary spongiosa. In vitro, the bone marrow macrophages from *Siglec15* null mice failed to form multinucleated mature osteoclasts [30, 31]. These phenotypes resembled those of *Tyrobp* null (i.e., DAP12-deficient) mice [32, 33], implying that Siglec-15 may be a primary DAP12-associated receptor involved in osteoclast differentiation in mice.

These findings also implied that Siglec-15 may be a therapeutic target for the osteoclast-mediated diseases. Takahata’s group found that *Siglec15* null female mice are resistant to osteoporosis induced by ovariectomy (i.e., estrogen deficiency) [34]. Although Siglec-15 was localized intracellularly in human myeloid cells in lymph node and spleen [17], it is expressed on the cell surface of osteoclasts [28, 29, 31, 35], allowing antibody-mediated therapeutic targeting. Tremblay and colleagues explored this possibility by developing monoclonal antibodies against Siglec-15 and demonstrating that in vivo administration of the antibody inhibited osteoclast differentiation and increased bone mass in healthy young mice [35]. Similar findings were reported by Takahata’s group using healthy young rats [36]. Taken together, these works suggested that Siglec-15 may be a therapeutic target for osteoporosis. Takahata and colleagues also reported that Siglec-15 may play a role in bone destruction in antigen-induced arthritis of mice (a model of rheumatoid arthritis), but not in joint destruction [37].

How does Siglec-15 modulate osteoclast differentiation? Siglec-15 appears to enhance phosphorylation of some key signal transducers, such as serine/threonine kinases ERK and AKT and phosphoinositide 3-kinase (PI3K), downstream of RANK–TRAF6 pathway [31, 35]. Thus, Siglec-15•DAP12–Syk pathway appear to cross-talk with RANK–TRAF6 pathway (Fig. 1a). How exactly this leads to altered osteoclast gene expression is not yet understood, as Siglec-15 deficiency does not influence the transcriptional regulation by NFATc1 [29, 31] or NFκB [31], two key transcriptional regulators of osteoclast differentiation. It is also worth noting that, although pathologic osteoclastogenesis induced by TNFα (a homolog of RANKL, signaling through TNFR–TRAF2 pathway) is also impaired in *Siglec15* deficient mice, ERK/PI3K/AKT phosphorylation in the TNFα-induced osteoclasts appear to be unchanged [34], implying the presence of yet unknown signaling pathway modulated by Siglec-15. How Siglec-15 modulates cytoskeletal rearrangement (actin ring formation) in osteoclast is also not understood.

Although the results from in vivo rodent models and in vitro human cell culture models are convincing, the involvement of Siglec-15 in human osteoclast differentiation in vivo has not been formally demonstrated. A study to show the association of a *SIGLEC15* polymorphism or deficiency with osteoclast-mediated human pathology, as was the case with *TREM2* deficiency (which causes polycystic lipomembranous osteodysplasia with sclerosing leukoencephalopathy, aka Nasu-Hakola disease [25, 26, 38]), is awaited.

### Siglec-15 in tumor immunity

Given that Siglec-15 recognizes sialyl-Tn structure [17], which is a well-known tumor-associated carbohydrate antigen [39], and macrophages play major roles in tumor immunity [40–42], it appeared logical to ask whether Siglec-15 is expressed on tumor-associated macrophages and plays a role in the tumor microenvironment. We found that Siglec-15 is induced by M-CSF (a cytokine inducing alternative activation/polarization of macrophages), and is expressed on tumor-associated macrophages [43]. Co-culture of sialyl-Tn^+^ cancer cell line and M-CSF–induced human macrophages or Siglec-15^+^ myeloid cell line enhanced the myeloid cell production of TGF-β (a pleiotropic cytokine that promotes epithelial-mesenchymal transition and metastasis of cancer cells) [43], which was dependent on DAP12 and SYK. These findings suggested that Siglec-15 may play a role in tumor microenvironment, but in vivo proof was lacking.

A recent study by Lieping Cheng’s group revealed a role of Siglec-15 in tumor [44]. They showed that Siglec-15 protein suppressed T cell proliferation and activation in vitro, which was verified in vivo using Siglec-15 deficient mice. T cell suppression appears to depend on IL-10, although whether IL-10 is produced by myeloid cells or T cells was not addressed. They also found that Siglec-15 is expressed on tumor cells and/or tumor-associated stromal cells (including tumor-associated macrophages) in non-small cell lung carcinoma clinical samples. In a mouse melanoma model (B16 cell line over-expressing GM-CSF, a cytokine involved in myeloid cell recruitment to tumor), Siglec-15 deficiency promoted T cell responses, better tumor control and overall survival. Siglec-15 targeting with monoclonal antibody in wild-type mice reversed the T cell suppression, attenuating cancer growth. In this disease model, Siglec-15 plays a role as a “ligand” for an unknown inhibitory receptor on cytotoxic T cells, in much the same way as PD-L1 (aka B7-H1, CD274) on cancer cells or tumor stroma engages immune checkpoint molecule PD-1 on T cells (Fig. 1b) [45–47]. Of note, although Siglec-15 does not show particularly close similarity with “B7 family” of immunoregulatory molecules, the expression of Siglec-15 (which was suppressed by interferon-γ) was inversely correlated with that of PD-L1 (which was induced by interferon-γ), implying that Siglec-15 targeting may be a complementary approach for the cancer patients who are refractory to PD-1/PD-L1–targeting therapies [44]. Whether sialic acid is required for the interaction between Siglec-15 (on cancer cells or stromal cells) and its “receptor” on T cells in tumor microenvironment is an open question. In this respect, a recent report on the suppression of cancer cell phagocytosis by macrophages via interaction between CD24 and Siglec-10 (on cancer cells and tumor-associated macrophages, respectively) may provide an insight. This study demonstrated that CD24–Siglec-10 interaction apparently does not require sialic acids, while the removal of sialic acids from cancer cells also enhances phagocytosis by macrophages independent of CD24 [48]. Thus, as glycan-independent Siglec function via protein–protein interaction is possible, a careful study would be required to tease apart glycan-dependent and -independent components in Siglec-15 functions.

### Siglec-15 in infectious diseases

In addition to the role of Siglec-15 in tumor microenvironment, two recent papers revealed the potential role of Siglec-15 in microbial infections. First, a multi-modal analysis of recurrent vulvovaginal infection by *Candida albicans* (including whole exome sequencing of European females, 155 cases and 172 controls) revealed that a *SIGLEC15* polymorphism (rs2919643 C, Phe273Leu) is a risk allele for the phenotype. Peripheral blood mononuclear cells (PBMCs) from donors with the risk allele, upon incubation with *C.albicans,* produced more T cell cytokines (e.g., IL-17, IL-22, and interferon-γ) than those from the donors without risk allele. The authors also found that human blood myeloid cells and a human vaginal epithelial cell line (in vitro), as well as mouse vaginal epithelial cells (in vivo), upregulate Siglec-15 mRNA upon *C.albicans* stimulation. These results imply that *C.albicans* induce Siglec-15 expression on myeloid cells (and/or epithelial cells), which in turn modulates T cell activity, a pattern that resembles how Siglec-15 operates in tumor microenvironment (Fig. 1b). As Phe273 is located adjacent to the Lys274 interacting with DAP12, this polymorphism may influence the signal transduction by Siglec-15. The authors showed that Siglec-15 directly binds *C.albicans*, and pre-treatment of *C.albicans* with sialidase altered the responses (reactive oxygen and cytokine productions) of PBMCs from healthy donors [49]. In this regard, although the presence of sialic acid on *C.albicans* have been reported [50], the genome of *C.albicans* does not appear to contain the homologs of the genes involved in the biosynthesis of sialic acid in bacteria and deuterostomes [51]. Sialic acid may be synthesized by a unique mechanism or acquired from the environment by *C.albicans*.

Another recent association study (involving 114 pairs of pulmonary tuberculosis patients and their asymptomatic household contacts in West Bengal, India) showed that another *SIGLEC15* polymorphism (rs61104666 A, synonymous substitution at Glu292) is associated with pulmonary tuberculosis [52]. The influence of this polymorphism on Siglec-15 protein is unknown, while it appears to be in linkage disequilibrium with the SNP rs2919643 in Europeans [49] and many other non-African populations (according to 1000 Genomes data). Whereas *Mycobacterium tuberculosis* is not known to express sialic acids, if *M.tuberculosis* induces the expression of Siglec-15 on myeloid cells, which in turn modulate T cell responses, this genotype–phenotype association may be explained by a similar mechanism implied for cancer immunity and *Candida* infection (Fig. 1b). In addition, *M.tuberculosis* infection of macrophages causes the formation of giant multinucleated cells called granuloma. If the granuloma formation is mediated by macrophage fusion (as traditionally assumed, which is now challenged [53]), Siglec-15 might participate in this process as it does in osteoclast fusion.

### Siglec-15 ligands

The involvement of sialic acids in osteoclast differentiation was demonstrated by Takahata’s group even before Siglec-15 was found [54]. To identify the sialylated glycoprotein on osteoclast precursors serving as a ligand for Siglec-15, we developed a method to introduce biotin label into the protein ligands of Siglecs using tyramide radicalization principle [55]. In brief, cells that express Siglec-15 ligand are incubated with a recombinant Siglec-15 probe coupled with peroxidase, which generates short-lived biotin-tyramide radical that reacts with tyrosine residue in the vicinity to yield a stable adduct. Using this method, we identified CD44, a heavily glycosylated protein, as a ligand for Siglec-15 on RAW264.7 cells. Knockdown of CD44 in RAW264.7 cells reduced the Siglec-15 binding and attenuated cell fusion. This finding also implies that CD44 may be a cancer cell-associated ligand for Siglec-15, as CD44 is highly expressed on many types of solid tumor [56, 57]. However, whether CD44 is a T cell ligand (or rather, “receptor”) for Siglec-15 in tumor microenvironment is unknown.

As mentioned above, although sialyl-Tn (Fig. 2a) is a preferred ligand for Siglec-15 [17], the glycan probes used in the study was limited. We therefore attempted to expand the repertoire of glycans to be probed, in collaboration with Dr. Chun-Cheng Lin (National Tsing Hua University) [18]. We observed decent binding of Siglec-15 to oligosaccharide Neu5Acα2–3[Neu5Acα2–6]Galβ1–4Glc/GlcNAcβ1- (Fig. 2b), whose presence in mammals has not been reported. Curiously, this oligosaccharide was also a good ligand for some other Siglecs (Siglec-7/9/14) [18]. Although biological significance of this finding is unknown, it was reported that sialylated and sulfated oligosaccharide (Neu5Acα2–3[HSO_3_–6]Galβ1–4GlcNAcβ1-; Fig. 2c) resembling these oligosaccharides is a preferred ligand for Siglec-8 [19], and such structure may be present on keratan sulfate in cartilage [58]. It would be of interest to test whether this sialylated and sulfated glycan structure is recognized by Siglec-15. Thus, the exact structure of biologically relevant glycan that is preferentially recognized by Siglec-15 is still not fully understood.

The glycans preferentially recognized by Siglec-15 were also sought with a novel approach called “cell-based glycan array” by Macauley and Wu [20]. They introduced a sialic acid derivative with an alkyne group (i.e., C5-substituted with N-propargyloxycarbonyl group) by sialyltransferases (ST6Gal-I or ST3Gal-IV) into the cell surface glycoconjugates of a sialic acid-deficient cell line. The sialic acid structures were diversified with a library of small chemical compounds with azide group by click chemistry. They found some sialic acid derivatives were particularly good ligands for Siglec-15 (Fig. 2d) [20]. (A similar approach was also developed by another group [59, 60], but Siglec-15 was not screened in their studies.) Together, these studies demonstrated how chemical biology can guide the discovery of specific and high-affinity inhibitor for Siglec-15 and other Siglecs. Further studies to identify the glycan structure(s) preferentially recognized by Siglec-15, as well as structural diversification of such glycans in combination with structure–activity relationship analysis, may eventually lead to potent Siglec-15 inhibitors with translational potential.

## Conclusion

Exciting new studies revealed the biological roles of Siglec-15 not only in osteoclast differentiation but also in tumor microenvironment and microbial infections. Although the exact mechanism by which Siglec-15 regulates tumor immunity and microbial infection is incompletely understood, the published data appear to imply that Siglec-15 may engage some protein “receptor” on T cells and dampen T cell responses (Fig. 1b). Future study to reveal the interacting partner on T cells for Siglec-15 with chemical biology tools would further advance our understanding of how Siglec-15 works, and how to utilize this knowledge for therapeutic gain.

## Data Availability

Not applicable.
